# Intra-Trackway Morphological Variations Due to Substrate Consistency: The El Frontal Dinosaur Tracksite (Lower Cretaceous, Spain)

**DOI:** 10.1371/journal.pone.0093708

**Published:** 2014-04-03

**Authors:** Novella L. Razzolini, Bernat Vila, Diego Castanera, Peter L. Falkingham, José Luis Barco, José Ignacio Canudo, Phillip L. Manning, Àngel Galobart

**Affiliations:** 1 Institut Català de Paleontologia Miquel Crusafont, Carrer de l’Escola Industrial, 23, Sabadell, Barcelona, Catalonia; 2 Grupo Aragosaurus-IUCA, Departamento de Paleontología, Facultad de Ciencias, Universidad de Zaragoza, Zaragoza, Spain; 3 Department of Comparative Biomedical Sciences, Structure and Motion Laboratory, Royal Veterinary College, London, United Kingdom; 4 Department of Ecology and Evolutionary Biology, Division of Biology and Medicine, Brown University, Providence, Rhode Island, United States of America; 5 Paleoymás S.L. Pol. Empresarium, Zaragoza, Spain; 6 Department of Earth and Environmental Science, University of Pennsylvania, Philadelphia, Pennsylvania, United States of America; 7 School of Earth, Atmospheric and Environmental Sciences, University of Manchester, Manchester, United Kingdom; Raymond M. Alf Museum of Paleontology, United States of America

## Abstract

An ichnological and sedimentological study of the El Frontal dinosaur tracksite (Early Cretaceous, Cameros basin, Soria, Spain) highlights the pronounced intra-trackway variation found in track morphologies of four theropod trackways. Photogrammetric 3D digital models revealed various and distinct intra-trackway morphotypes, which reflect changes in footprint parameters such as the pace length, the track length, depth, and height of displacement rims. Sedimentological analyses suggest that the original substrate was non-homogenous due to lateral changes in adjoining microfacies. Multidata analyses indicate that morphological differences in these deep and shallow tracks represent a part of a continuum of track morphologies and geometries produced by a gradient of substrate consistencies across the site. This implies that the large range of track morphologies at this site resulted from similar trackmakers crossing variable facies. The trackways at the El Frontal site present an exemplary case of how track morphology, and consequently potential ichnotaxa, can vary, even when produced by a single trackmaker.

## Introduction

Track morphology is determined by both the trackmaker and the substrate characteristics [1]–[4]. Although it is widely accepted that the substrate is a major control in determining the final track morphology [1], [2], [5]–[7], studying this dynamic formation process is challenging given the fact that most foot-sediment and sediment-sediment interactions are highly complex, rapid and hidden from view [8]–[10]. Baird [11] stated that a trackway is not a simple record of anatomy; instead, it is a record of how a foot behaves under a distinct locomotory pattern as it makes contact with a particular substrate. The way in which sediments behave before, during, and after a track is formed and the subsequent processes that may further modify, enhance, or disguise a track has been much neglected [1], [12]. Hence, to understand the formation and preservation of tracks, it is essential to understand the mechanics of soils and rheology [13]–[15].

Traditionally, ichnology has primarily studied tracks and trackways as two-dimensional traces (e.g., [16], [17]), rarely considering the substrates mechanics and prevailing condition at the time a track-maker’s foot made contact with a sediment. For ichnological analyses to be well founded, footprints must be documented by methods that avoid inaccurate representations of track morphology, which can distort or obscure potentially important data [18]. Recent advances agree that the foot’s contact with a substrate can only be understood by taking a three-dimensional approach to explain track formation [1], [5], [19]–[21]. The variation in track morphology due to sediment consistency can be observed and quantified through the use of three-dimensional (3-D) technologies (i.e. using laser scanning or photogrammetry to show depth analyses and vertical cross sections) [22]–[25] with the intention to integrate quantitative analytical techniques with the traditional ichnotaxonomic definition. Light Detection And Range (LiDAR) techniques [22], [23], [26] together with photogrammetry methods [27] complement the classic ichnological data acquisition by providing accurate data on 3-D specimens.

The present study concentrates on the quantification of morphological variability of tridactyl dinosaur tracks documented at the El Frontal tracksite (Lower Cretaceous, NW Iberian Peninsula), which were briefly mentioned in previous works [28]–[31] but never studied in detail. The aim of this work is to quantify the inter- and intra-trackway morphological variation *sensu* [10] recorded in different track shapes to underpin the variability in track morphology when track-maker is kept constant. This study will focus on four long trackways that are characterized by a range of track morphologies that are considered as indicators of rheological conditions.

## Geological Setting

**Figure 1:**
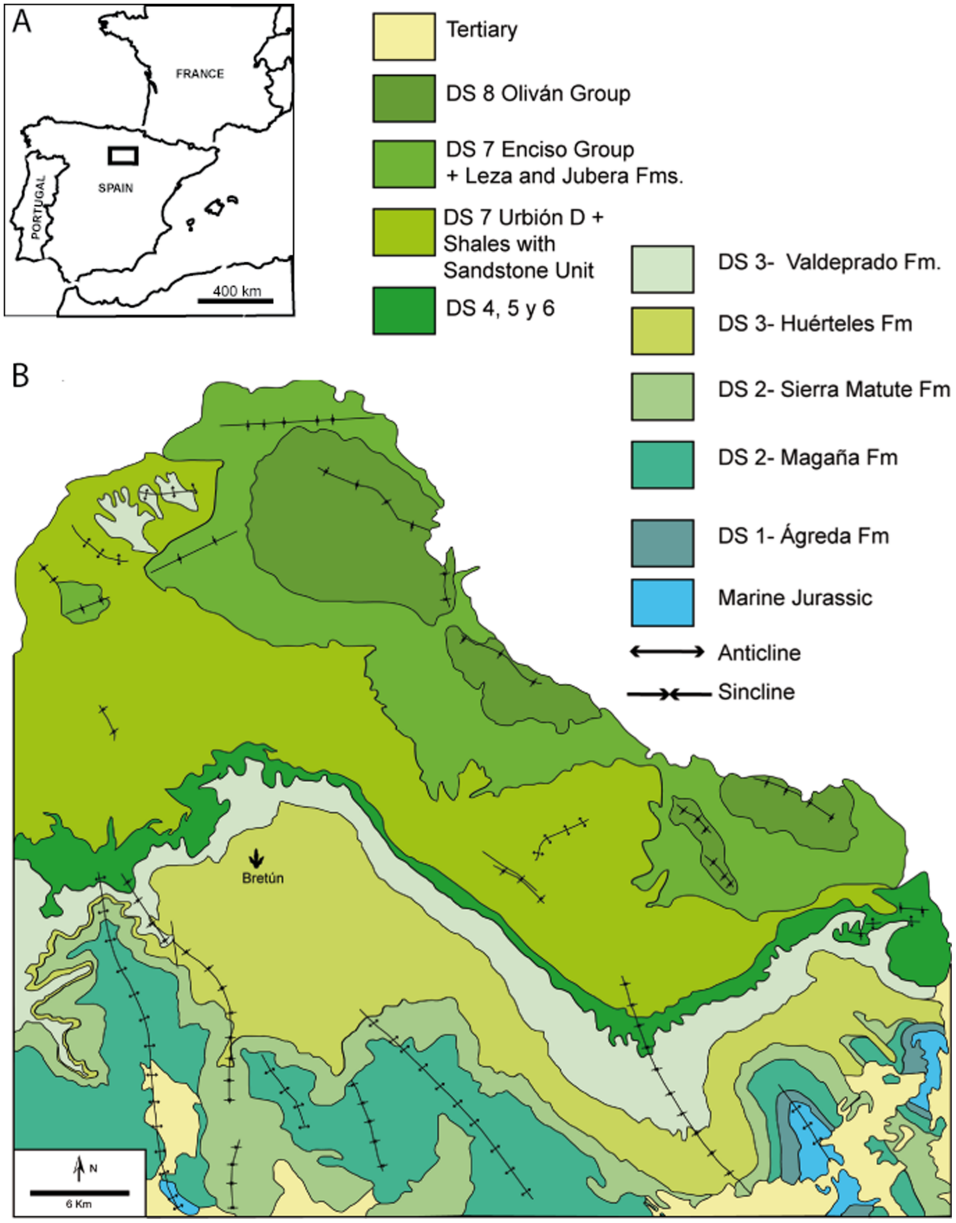
Geographical and geological setting of the El Frontal tracksite (Bretun, Soria). The location of Bretun locality within the Iberian Peninsula is inside the black square. The tracksite locates in DS-3 of the Huerteles Fm [32]. Geological map modified from Castanera et al. [33].

The El Frontal site is found in the Cameros Basin (Soria, Spain), which is located northwest of the Iberian range. The sedimentary infill of the Cameros basin was divided in eight depositional sequences, with deposits predominantly from continental environments [32] (Fig. 1). The sedimentation was dominantly continental as demonstrated by alluvial and lacustrine deposits [34], but includes some sporadic marine incursions [35]–[38]. The tracksite falls in the DS-3 (depositional sequence, Fig. 1) and belongs to the Oncala Group. It is subdivided into the Huérteles (which includes the El Frontal tracksite) and Valdelprado formations and dates to the Berriasian [32], [35], [39], [40]. The depositional sequence DS-3 follows a pattern of alluvial fans and lacustrine sediments that thin laterally (northwesterly) to fluvial and fluvio-lacustrine deposits [32]).

Recent interpretation describes the Huérteles Formation as characterized by terrigenous sediments (fluvial system) in the western sector of the eastern Cameros Basin and by an increase in shallow, coastal, carbonate-sulphate water bodies to the east, implying that the connection of the Cameros basin with marine areas was much stronger [38] than previously considered [34]. A series of sedimentary structures that crop out near the El Frontal tracksite, (i.e. inclined heterolithic stratification, flaser, rhythmic alternations of sandstones and lutites, symmetrical ripples, mud-drapes) are indicative of a tidally-influenced fluvial-deltaic environment [37], [38].

**Figure 2:**
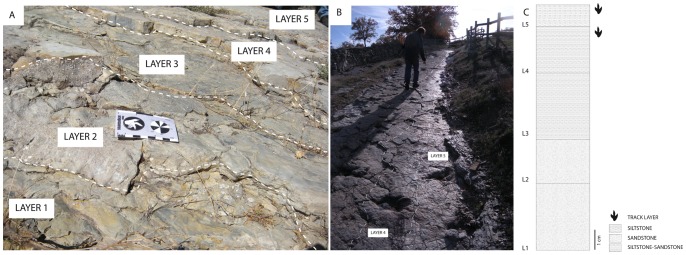
Tracksite microlayers organization. A) The El Frontal tracksite is composed of 5 different centimeter-thick layers that intercalate gray siltstones, limestone and sandy-siltstones. Scale bar equals 8 cm. B) El Frontal track layers 4 (penetrative tracks) and 5 (tracking surface), where all the studied tracks originated. When thin layer 5 is not preserved, tracks are found in level 4. C) Stratigraphical log of the five layers found in the El Frontal tracksite. Theropod tracks are found in layers 5 and 4. Scale bar equals 1 cm.

**Figure 3:**
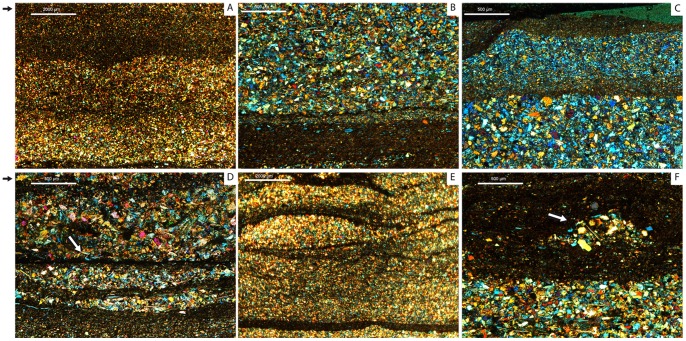
Thin sections IPS-82477a-d of layers 4 and 5 of the El Frontal tracksite. A) Sandstone intercalated by siltstone-mudstone bands in which chlorite minerals are scarce (<5%); B) High quartz concentration (>60%) and scarce presence of clay minerals in mud bands; C)Sandstone-siltstone in which the grain size decreases from the bottom to the top; D) Sandstone intercalated by siltstone, mineral clays are abundant (>60%); E) Deformation structures (mud drapes); F) Deformation structure (symmetrical ripple). Black arrows indicate the top of the laminae, white arrows indicate deformation structures. Scale bars are 2000 μ for A, 500 μ for B, C, D, F and 2000 μ for E.

The El Frontal tracksite is 150 meters apart from the outcrops of the Fuente Lacorte tracksite reported by Aguirrezabala and Viera [28] and Sanz et al. [29]. The latter is stratigraphically lower with respect to the studied locality. The lithology of the El Frontal tracksite is composed of 5 different layers that include intercalated organic rich gray siltstones mudstones and sandy-siltstones (Fig. 2A–C). In detail, trackways and isolated tracks are produced and impressed in layer 5 (tracking surface). Layer 5 is a 1 cm-thick siltstone with occasional mud cracks (Fig. 2C) which sometimes is not preserved, and tracks and trackways are found as undertracks in the underlying layer, layer 4. This is characterized by a 2–3 cm thick sandstone-siltstone (Fig. 2B–C). The first set of laminas (Fig. 3A–C) observed at different areas of the tracksite reveals that layer 4 and 5 are composed of quartz (>60%), and minor abundance of phyllosilicates, and chlorite minerals (Fig. 3A–C). It has a grain-supported fabric with quartz ranging from fine to medium size, yielding a moderately sorted composition. The chlorite minerals (<5%) and other planar minerals are very scarce in the mudstone band in the clay matrix. The second set of laminas including layer 4 and 5 was collected not far from the first (Fig. 3D–F) and is composed of sandstone intercalated with mudstone. In these laminas, sedimentary structures, such as mud drapes (Fig. 3D, 3E) and symmetric ripples (Fig. 3F) are observed. These are characterized by a higher percentage of chlorite minerals (>60%) that concentrate in the mudstone band in the clay matrix. Mud drape structures form when a sediment undergoes intermittent flows, leading to alternating sand and mud layers [37]. Symmetrical ripples are formed when a horizontal oscillation generates wave ripples formed by rolling grains in shallow water [41], and they are commonly found associated with mud drapes [37].

## Materials and Methods

A complete digital model of the track-bearing outcrop was generated using a RIEGL LMS-Z420i long range 3D laser scanner capable of 5–10 mm resolution [22], [23], [42] (for three-dimensional El Frontal tracksite caption see Appendix S1). The three-dimensional surface of the tracksite El Frontal is available as a polygon file in the Supplementary Information. This overview scan was complemented with close-range photogrammetric models [27] of individual tracks (Appendix S2), produced from 10 to 20 photographs per track and processed using VisualSFM (http://ccwu.me/vsfm/) [43].

**Figure 4:**
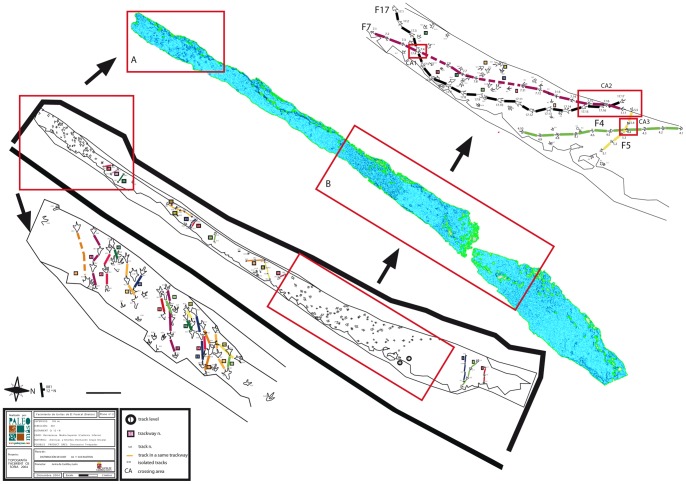
Cartography and 3-D model of the El Frontal tracksite resulting from the LiDAR scanning (grey colour), modified from Barco et al. [44] and designed by Paleoymas SL. In the red rectangles (A and B) are the details of the areas with the highest density of tracks. The studied area is detailed in the rectangle B. Studied trackways F17, F7, F5 and F4 are coloured respectively with black, pink, yellow and green.

**Figure 5:**
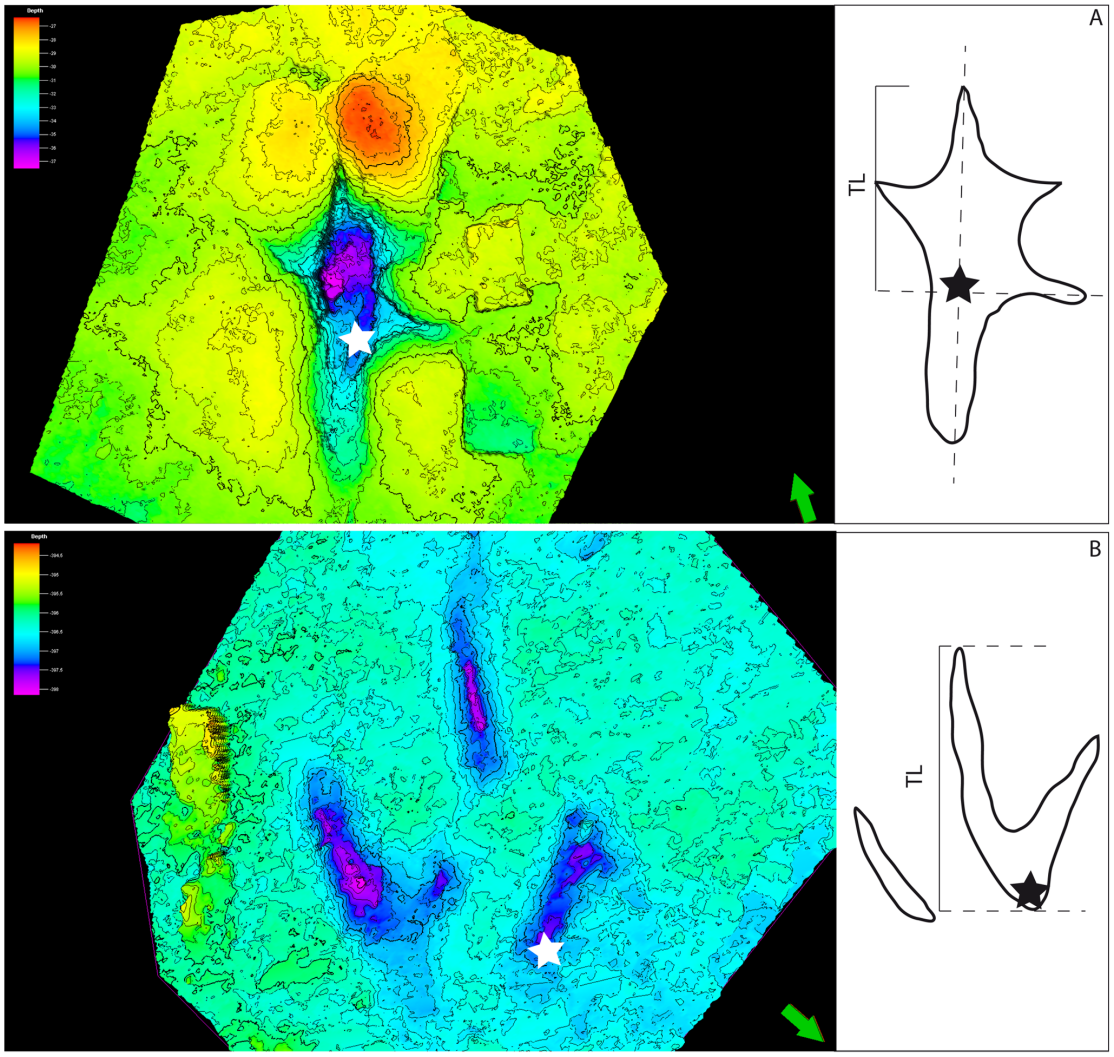
Standards for measuring track length (TL) and depths (black and white stars). A) TL excludes the elongated metatarsal impression, and the depth is taken approximately were the phalanx 1 of metatarsal IV should be. B) In this case there is no metatarsal impression and the TL is easier to measure. Depth is taken in the same point for every track. Color scale green indicates the track layer, purple is the deepest point recorded and red is the highest point recorded.

Four trackways (F17, F7, F5, and F4) spanning the site were studied in detail, comprising a total of 49 tracks (17, 17, 5, and 10 tracks from the respective trackways) (Fig. 4). These trackways were chosen for their high morphological variability and their proximity to each other, with the aim of reflecting any effect of spatial variation in substrate consistency. For each track, several metrics were measured from the photogrammetric models using both ImageJ software and Schlumberger package Petrel: track length (TL, measured from tip of digit III, excluding metatarsal pad when present), track width (TW), interdigital angles (II∧III, III∧IV, II∧IV), displacement rim height (DR), maximum depth (D_max_), and depth of the metatarsal pad impression (D_mp_). The two depth metrics were recorded, because many of the tracks show signs of post-formational sealing of the track walls around the digit impressions. Maximum depth is therefore interesting to note, but is of no use for comparisons between tracks (though it remains a useful metric in tracks where no sealing has occurred). The metatarsal pad, conversely, rarely suffers from such wall collapse due to the width of the impression, and so depth recordings from this homologous point between tracks can be comparatively informative. Unfortunately, the metatarsal pad is not always impressed. However, by recording both depth metrics where possible, an indication of the track morphology can be conveyed (Fig. 5A–B). Additionally, pace length (PL) and stride length (SL) were measured both in the field and using the whole-outcrop digital model. Statistical analyses on the 49 tracks refer to linear correlation and dispersion plots that interpolate track length (TL), depth (D) and displacement rim height (DR) parameters.

To quantify the substantial intra-trackway depth and length variations, four graphs for trackways F17, F7, F5 and F4 were built using TL, PL (left Y axis) and depth measurements (right y axis). A sedimentological analysis (4 thin sections in total, IPS-82477a-d housed at the Intitut Català de Paleontologia “Miquel Crusafont”-ICP) for layers 4 (undertracks) and 5 (tracking surface) was undertaken to quantify lithology and mineral composition of the sediment. Pictures of the four polished thin sections(Fig. 3A–F) were taken using light microscopy via a Leica DM 2500 photo-microscope.

## Results

The El Frontal tracksite consists of a southwest-northeast orientated outcrop containing more than 200 tridactyl tracks and 45 trackways (see Table 1) [28]–[31], distributed along 185 m^2^ surface area (Fig. 4). Track density is of more than one track/m^2^, although tracks are not homogenously distributed (Fig. 4A–B).

**Table 1:** Table with measurements taken for all the theropod trackways in the El Frontal.

TRACK	TL	TW	II∧III	III∧IV	II∧IV	PL	SL
**45.1**	34.4	15.6	23.6	18.4		85.4	166.1
**45.2**	34	x	x	x		85.1	x
**45.3**	32.2	18.7	22.8	26.3		x	x
**44.1**	19.2	17.4	24.2	38.7		68.8	115.7
**44.2**	31.3	20	24.8	19		54.1	108.2
**44.3**	27.8	16.8	17.2	20.9		62	x
**44.4**	20.1	15.8	24.7	21.3		x	x
**43.1**	26	18.5	21.4	42.9		62.2	124.9
**43.2**	26	17.7	20.3	30		66	x
**43.3**	21	18.5	25.3	22.5		x	x
**42.1**	31	18.1	x	x		65.4	x
**42.2**	26.4	15	x	x		x	x
**41.1**	39	24.3	22.2	19.3		71.4	133
**41.2**	27.2	14.3	19.7	17		66.5	x
**41.3**	22	19.2	20.4	16.5		x	x
**40.1**	21	23.7	29.6	34.1		66.1	x
**40.2**	25	20.6	32.3	38.6		x	x
**39.1**	22.1	16.5	35.7	19.24		71.1	x
**39.1**	22.4	22.4	25.6	17.44		x	x
**38.1**	22.7	17.8	17	14.6		74.1	x
**38.2**	22	x	x	x		x	x
**37.1**	18.1	15.5	32.5	39.2		66.1	150.5
**37.2**	14.6	18	24.4	44		82.3	139.1
**37.3**	24.7	18.1	27.6	22.5		54.1	x
**37.4**	14.7	18.7	54.2	51.37		x	x
**36.1**	21	16.5	13.7	25.6		39.6	x
**36.2**	19.6	15.8	28.05	39.3		x	x
**35.1**	19.3	22.1	16.4	18.4		52	x
**35.2**	21.7	15	x	x		x	x
**34.1**	25	14	25.2	30.8		87.5	x
**34.2**	26.2	19.8	17.4	25.6		x	x
**33.1**	23.4	19.4	38.8	36.3		90.3	163.7
**33.2**	23.4	27.1	21.3	25.4		69.1	x
**33.3**	23.7	16.3	x	x		x	x
**32.1**	15.5	15.3	x	25.2		70.8	146.3
**32.2**	23	19.3	19.8	24.4		76.2	x
**32.3**	23.6	19	28.9	30.8		x	x
**31.1**	21.4	18.2	33.3	18.6		67.1	131
**31.2**	26.2	23.4	35.8	35.1		76.1	x
**31.3**	22.7	15.5	27.5	29.8		x	x
**30.1**	17.8	13.5	54.7	x		62.9	x
**30.2**	19.6	18.8	24.2	26.5		x	x
**29.1**	18.3	21.7	26.3	28.3		86.1	x
**29.2**	20.4	18.7	18.5	17.6		x	x
**28.1**	5.8	5.3	29.5	30.3		28	x
**28.2**	5.2	4.6	24.9	32.3		x	x
**27.1**	7.8	6.4	21.07	30.6		25.1	x
**27.2**	7.7	7.8	35.5	32.7		x	x
**26.1**	6	5	26.5	26.7		28.6	x
**26.2**	5.1	6.2	23.52	30		x	x
**25.1**	21	27	68.2	77.6		97.2	202
**25.2**	28.4	21.8	45.3	36.5		107	x
**25.3**	28.4	20.2	43.8	36.5		100.6	x
**25.4**	25.2	19	40.2	42.5		x	x
**24.1**	6.5	5.3	24.9	26.8		26.8	x
**24.2**	5	5.5	40.8	28		x	x
**23.1**	5.5	6.3	31.2	39.1		16.1	29.5
**23.2**	6.9	5.8	27.2	39.2		15.3	x
**23.3**	3.8	5	42	42		x	x
**22.1**	5.8	5	20.5	20.1		23.7	x
**22.2**	5	5.6	25.3	25		x	x
**21.1**	7	6.3	36.7	28.5		26.3	x
**21.2**	7	6.1	12.9	26.1		x	x
**20.1**	8.2	5.4	20	27.1		27.4	53.5
**20.2**	6.4	5.9	30.4	36.8		26.3	x
**20.3**	5.4	4.3	40.2	47		x	x
**19.1**	4.6	4.4	60.2	43		18.4	34.6
**19.2**	4	4.1	34.4	30		17.2	35
**19.3**	4.2	3.5	52.3	30.5		18	x
**19.4**	5.3	4.8	32.4	40.8		x	x
**18.1**	4.6	4.4	17.7	28.6		x	x
**18.2**	7.3	7.9	55.7	49.1		20.1	x
**17.1**	30	14.7	27.3	25.6		91	180
**17.2**	29	13.2	22.1	19.9		95	182
**17.3**	27	17.7	26.7	32.7		96	177
**17.4**	31	14.0	19.7	22.1		85	175
**17.5**	29	14.3	21.5	30.3		90	
**17.6**	27	11.876	30	37.2	76.8	100	195
**17.7**	26	12.881	28.4	25.1	68.9	94	193
**17.8**	32	13.333	31.6	31.3	54.2	98	213
**17.9**	27	14.465	24.6	28.6	57.5	115	222
**17.10**	32	14.165	24.7	26.6	46.3	108	193
**17.11**	34	14.712	26.4	27.3	48.3	88	178
**17.12**	31	13.535	18.9	26	52.3	98	200
**17.13**	30	16.311	22.6	27	48.4	103	198
**17.14**	29	16.078	19.8	25.8	51.4	100	198
**17.15**	32	14.307	32	35.7	54	100	197
**17.16**	27	12.819	27.3	30.9	68.8	94	
**17.17**	30	16.297				100	
**16.1**	10.9	10.3	46.7	36.5		33.5	62.4
**16.2**	12	9.1	29.8	42		30.2	61.8
**16.3**	12.2	10.9	37.9	35.7		33.9	x
**16.4**	12.3	10.6	36	35		x	x
**15.1**	8	9.1	35.6	30.6		48.8	x
**15.2**	10.3	8.7	28.7	42		x	x
**14.1**	12.7	10.2	26.7	36		44.8	x
**14.2**	13.4	9.8	30.4	32.1		x	x
**13.1**	15.5	12.1	23.9	38.8		40.4	81.2
**13.2**	15.7	12.1	22.8	29.1		42.4	x
**13.3**	15.5	14.6	20.1	33.2		x	x
**12.1**	18.3	11.8	29.1	32.2		44.8	91
**12.2**	18.8	11.8	33.2	20.2		48.8	95.2
**12.3**	16.2	11.4	26.2	21.6		49.4	x
**12.4**	20.5	10.8	27.5	35.1		x	x
**11.1**	12	11.3	42.7	34.4		62	119.4
**11.2**	13.4	11.8	32.8	31.3		58.7	119
**11.3**	13.8	10.5	32.9	34.6		60.8	x
**11.4**	13	11.3	30.7	33		x	x
**10.1**	18.2	15.2	25.4	38.4		50	x
**10.2**	16.4	14.3	31	35.2		x	x
**9.1**	12.3	10.2	30.4	45		48.6	92.4
**9.2**	14.2	10.2	34.4	33.5		44.6	93.1
**9.3**	14.2	11.5	30	40.4		49.6	x
**9.4**	11.7	11.3	31.9	44.2		x	x
**8.1**	8.9	9	28.5	46.6		36.1	80.6
**8.2**	8.7	9.3	36.6	47.1		45	68
**8.3**	11.3	9.3	48.7	48.7		24.9	x
**8.4**	10	8.8	38.6	46.5		x	x
**7.1**	20	11.0	29.2	27.9	57.6	88	185
**7.2**	21	13.9	26.3	39.6	59.3	98	193
**7.3**	23	13.9	27.3	42.4	65.9	100	
**7.4**	22	10.2	26.1	35.7	55.7	95	181
**7.5**	22	11.1	x			95	176
**7.6**	24	10.1	25	18.6	47.1	93	167
**7.7**	25	11.0	41.4	36.3	58.3	84	174
**7.8**	25	9.5	22.2	22	53.6	82	184
**7.9**	26	11.4				94	
**7.10**	22	10				93	196
**7.11**	25	11.2	45.9	17.2	59.6	103	201
**7.12**	24	13.0	27.9	29.6	51.3	102	195
**7.13**	21	10.7	32.9	26.2	66.7	99	193
**7.14**	22	12.0	30.6	31.7	59.6	97	197
**7.15**	25	10.9	38.3	52.6	64.7	100	195
**7.16**	19	10.7	32.5	38	73.4	99	
**7.17**	25	11.0				99	
**5.1**	17	12.0	29	41.4	77.7	74	142
**5.2**	19	12.1	31.6	48	78.6	68	134
**5.3**	16	8.7	39.4	43.2	80.3	69	135
**5.4**	18	10.4	28.5	39.7	70	66	134
**5.5**	22	11.7				65	
**4.1**	23	11.0	36.2	28.4	67.1	106	207
**4.2**	26	10.3	39.7	40.8	85.9	103	204
**4.3**	26	9.7	38.7	34.6	66.8	101	202
**4.4**	27	14.0	52.2	42.4	72.2	103	204
**4.5**	30	11.0	34	24.5	58.6	105	202
**4.6**	23	12.6	45	30.1	73.8	102	201
**4.7**	26	11.0	31.2	23	51.3	100	204
**4.8**	22	12.6	37.6	35	70.7	100	200
**4.9**	24	12.2	36.4	38.7	61.8	103	
**4.10**	27	12.3	43	31.5	70.2	100	
**3.1**	5.6	5.5	42.1	45.8		25.5	50.5
**3.2**	5.4	5.5	44.3	43.5		25.2	50
**3.3**	6.2	6.1	30.2	53.3		24.9	x
**3.4**	2.9	5.3	38	25.3		x	x
**2.1**	42.08	33.05	57.12	34.74		103	
**2.2**	38.18	39.13	61.8	44.25		113	210
**2.3**	46.87	41.1	51.03	51.25			
**2.4**							
**1.1**	7.5	6.8	32.2	36.6		22.8	44.9
**1.2**	8.6	6.6	34.6	37		21.8	x
**1.3**	7.3	5.6	25.2	37.3		x	x

TL (track length); TW (track width); II∧III (interdigital angel between II∧III); III∧IV (interdigital angle between III∧IV); II∧IV (interdigital angle between II∧IV taken for trackways 17, 7, 5 and 4); PL (Pace length); SL (Stride length). All measurements in Table 1 are in CM.

We describe the position in the tracksite, spatial distance and possible interaction of trackways F17, F7, F4 and F5 with one another. At the northeastern edge of the outcrop, the first tracks of trackway F17 are separated from those of trackway F7 by one meter (Fig. 4B). F17 crosses with trackway F7 (crossing area 1, Fig. 4B) at one meter from its origin. The crossing area includes tracks 17.4 and 7.4 (Fig. 4B), respectively, the former track overlapping the latter, and thus indicates the sequence of trampling. Trackway F17 turns east and aligns parallel to trackway F7 (Fig. 4B). They follow a north-northwest direction for about 10 meters. Trackway F17 finally crosses again with trackway F7 in a region that includes tracks 17.15 - 17.17 and 7.15 - 7.17 (crossing area 2, Fig. 4B). No overlapping of tracks is found in this area, although tracks are located very close to each other. Trackway F4 is parallel to but with an opposite direction to trackways F17 and F7, from which it is separated by 2 meters (Fig. 4B). Trackway F5 has a subperpendicular direction to trackways F17, F7 and F4, and intercepts trackway F4 at track 4.4 (crossing area 3, Fig. 4B) without evidence of overlapping.

### 1. Morphological Variation

**Figure 6:**
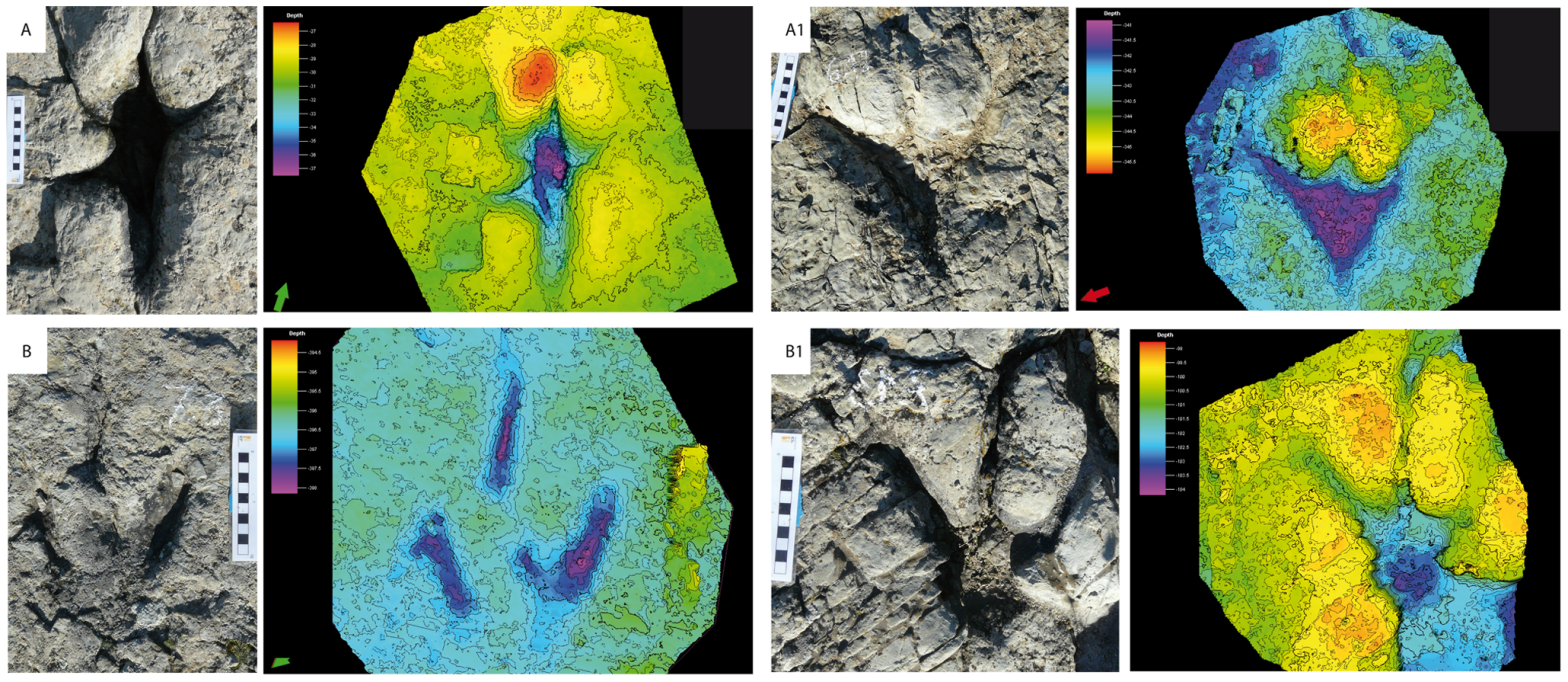
Morphological characterization of the El Frontal tracks. A) Morphotype A is track 17.2, A1) Variation of morphotype A, track 17.17, B) Morphotype B is track 7.3, B1) Variation of morphotype B, track 7.13. Color scale green and yellow indicates the track layer, purple is the deepest point recorded for depth and red is the highest point recorded for displacement rims.

Field observations and photogrammetric models (Appendix S2) revealed various intra-trackway morphotypes (Fig. 6). The morphological variation is exemplified by four different track shapes from the starting (17.2–7.3, Fig. 6A–B) and ending portions (17.17–7.13, Fig. 6A1–B1) of trackways F17 and F7. Track 17.2 in Figure 6A is characterized by being deep and poorly detailed with thin digital impressions (particularly Digit III), bounded by substantial displacement rims. In this regard, it is not uncommon to observe the exit hole *sensu* [45] p.39 of digit III. When digit III is long, and distinguishable, digits II and IV tend to be narrow due to wall collapse (e.g., tracks 17.1, 17.3, and 17.10; see three-dimensional model capture of El Frontal tracksite in Appendix S1). Conversely, when digit III is sealed and bounded by sediment ridges, impressions of digits II and IV are thicker (e.g., Tracks 17.17 and 5.2, Fig. 6A1, see Appendix S2). Track 17.2 (Fig. 6A) shows a deep central area and a deeply impressed and elongated metatarsal mark similar to that reported by Kuban [46]. Sometimes, tracks preserving impressions of digits II, III, and IV exhibit a posteromedially oriented hallux mark in the rear margin. Track 17.17 (Fig. 6A1) belongs to the same trackway as track 17.2, yet 17.17 lacks the hallux and metatarsal impressions which dominate the morphology of 17.2. On the other hand, track 7.3 (Fig. 6B) is a shallow track with a typical tridactyl appearance, digits II and IV usually well impressed, and digit III marked only in its distal part (eg., tracks 4.8 and 7.3, Fig. 6B, see Appendix S2 and Table 2). Track 7.3 shows very little extraneous substrate deformation. In the same trackway, track 7.13 (Fig. 6B1) is found, which differs substantially from 7.3, being considerably deeper and with displacement rims between digits II–III and III–IV. There is also a deep impression where the digits converge at the metarsal pad – an impression almost entirely absent from track 7.3. The tracks differ according to characters such as the presence/absence of hallux or metatarsus impressions, interdigital rims, mud collapse structures and pad impressions.

**Table 2:** Table with measurements taken for all tracks belonging to trackways F17,F 7,F 5 and F4 of the El Frontal tracksite.

TRACKS	D	TL	PL	DR
**7.1**	0,18	20	88	0.700
**7.2**	0,18	21	98	0.320
**7.3**	0,17	23	100	0.320
**7.4**	0,9	22	95	0.917
**7.5**	0,8	22	95	0.600
**7.6**	0,8	24	93	0.710
**7.7**	0,7	25	84	0.8
**7.8**	0,18	25	82	0.75
**7.9**	1,3	26	94	0.64
**7.10**	1,4	22	93	0.6
**7.11**	1,5	25	103	0.7
**7.12**	2,1	24	102	0.782
**7.13**	2,25	21	99	1.709
**7.14**	2,1	22	97	0.9
**7.15**	2	25	100	0.8
**7.16**	1,9	19	99	1.189
**7.17**	1,2	25	99	0.553
**average**	1,16	23	95,35	
**desvst**	0,74	2,06	5,97	
**max**	1,9	25,06	101,32	
**min**	0,41	20,94	89,38	
**17.1**	3,18	30	91	1.582
**17.2**	4,8	29	95	4.179
**17.3**	4,32	27	96	2.951
**17.4**	3,1	31	85	1.132
**17.5**	2,9	29	90	2.267
**17.6**	2,8	27	100	1.771
**17.7**	2,6	26	94	1.432
**17.8**	2,6	32	98	1.679
**17.9**	2,9	27	115	1.965
**17.10**	2,7	32	108	2.256
**17.11**	3,1	34	88	2.332
**17.12**	3,5	31	98	1.879
**17.13**	3,8	30	103	2.277
**17.14**	4,7	29	100	2.653
**17.15**	3,1	32	100	2.331
**17.16**	2	27	94	2.014
**17.17**	1,3	30	100	1.511
**average**	3,15	29,59	97,35	
**desv**	0,88	2,27	7,29	
**max**	4,03	31,85	104,64	
**min**	2,27	27,32	90,06	
**5.1**	2,2	17	74	1.1
**5.2**	2,2	19	68	1.1
**5.3**	1,7	16	69	0.365
**5.4**	1,4	18	66	0.2
**5.5**	0,8	22	65	0.398
**average**	1,66	18,4	68,4	
**desv**	0,59	2,3	3,51	
**max**	2,25	20,7	71,91	
**min**	1,07	16,1	64,89	
**4.1**	0,5	23	106	0.35
**4.2**	0,6	26	103	0.593
**4.3**	0,7	26	101	0.574
**4.4**	0,7	27	103	0.798
**4.5**	0,9	30	105	0.8
**4.6**	1	23	102	1.01
**4.7**	1,2	26	100	1.02
**4.8**	0,66	22	100	0.987
**4.9**	0,69	24	103	0.617
**4.10**	0,8	27	100	0.781
**average**	0,78	25,4	102,3	
**desv**	0,21	2,41	2	
**max**	0,98	27,81	104,3	
**min**	0,57	22,99	100,3	

DR (depth rims); D (depth of the track); TL (track length); PL (pace length). For each measurement average (media), standard deviation (desv) and values with the maximum and minimum standard deviation (max and min) are calculated. All measurements in Table 2 are in CM.

### 2. Quantification of Morphological Variation

**Figure 7:**
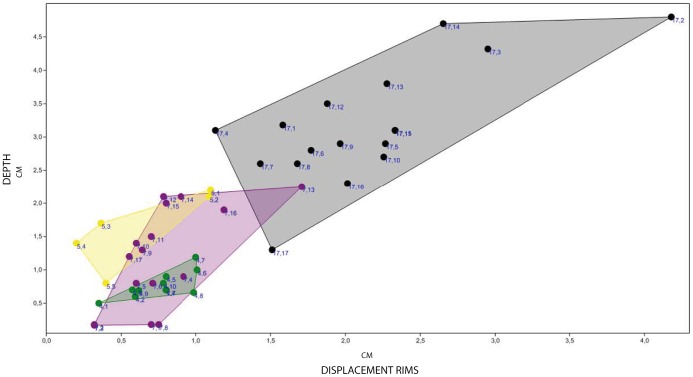
The linear correlation graphic of depth (D) vs displacement rims (DR) shows a positive correlation among these two values. Pearson’s correlation matrix results in r = 0.871 and Spearman’s correlation matrix in an r = 0.820. F17 (black colour), F7 (purple colour), F5 (yellow colour) and F4 (green colour). Units are in centimeters.

**Figure 8:**
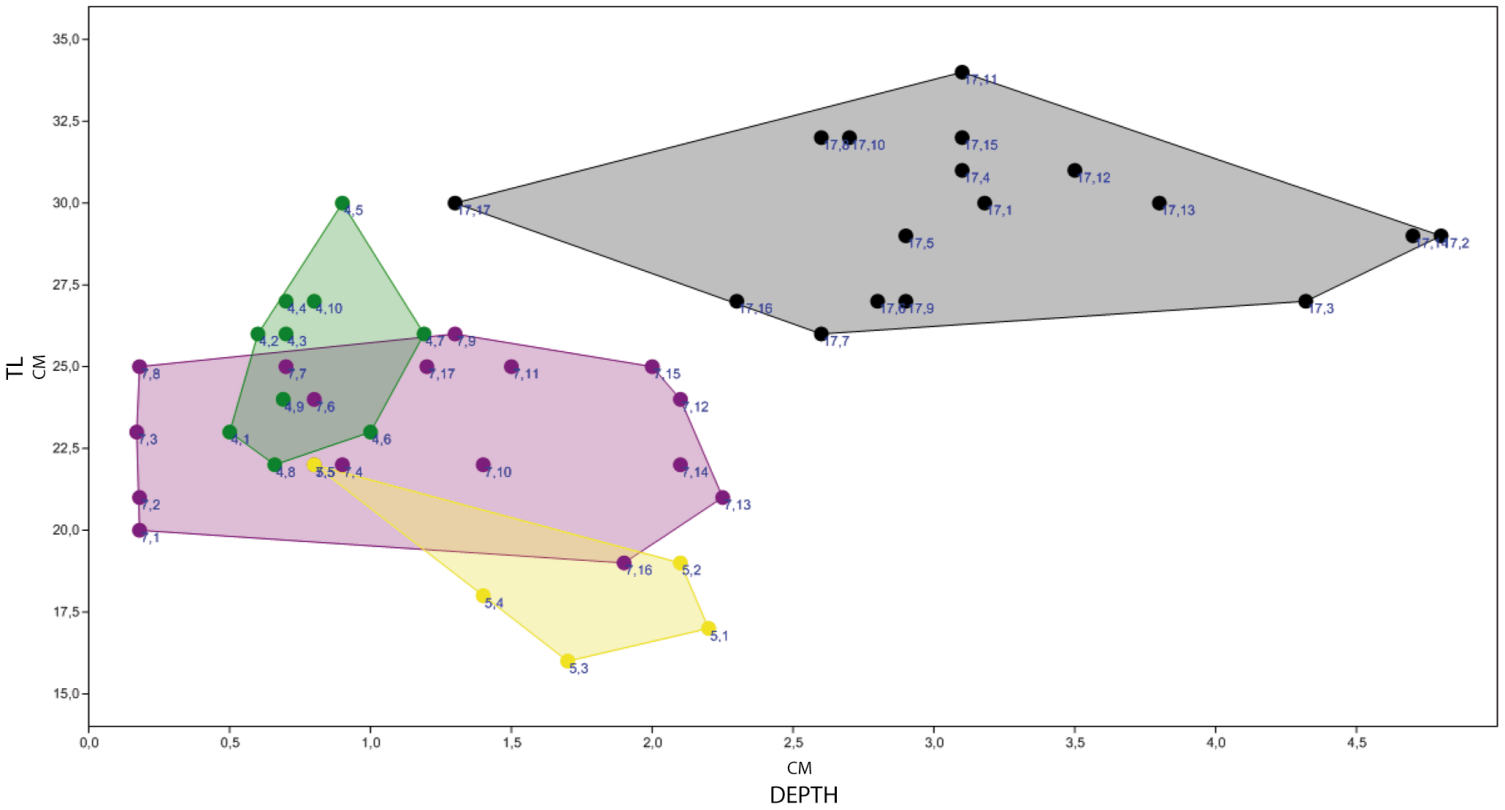
Dispersion graph of depth (D) vs track length (TL) shows a wide range distribution among the tracks of trackways F17, F7 and F5 (respectively black, purple and yellow colours) of the El Frontal tracksite. The most concentrated cluster in that of trackway F4 (green colour), in which values are quite consistent and only weakly vary along the trackway. Units are in centimeters.

**Figure 9:**
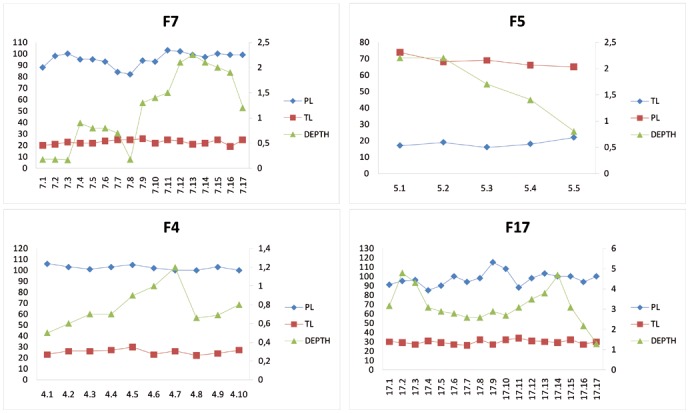
Quantification of the intra-trackway depth and length variations, four graphs for trackways F17, F7, F5 and F4 are built using TL, PL (left Y axis) and depth measurements (right y axis). Units are in centimeters.

The shape variation described above is reflected in changes in track parameters such as the measurable pace length (PL), track length (TL) and depth (D), and maximum height of the associated displacement rims (DR) (Table 2). This morphological variation is presented quantitatively in Figures 7–9.

The D versus DR graphic (see Table 2 and Fig. 7) shows the relationship between the depth (D) of the tracks, and the maximum sediment height of the associated displacement rims (DR) (Fig. 7). It shows that these two parameters are positively correlated (Pearson’s correlation matrix r = 0.871 and Spearman’s correlation matrix r = 0.820). Deeper tracks show the highest displacement rims between the digits.

The figure 8 shows considerable intra-trackway variation. More importantly, in deep tracks, measurable track length (TL) appears influenced by track depth. Thus, trackways F17, F7 and F5 show a wide range of values, displaying a very pronounced variability in both depth (D) and track length (TL) parameters (see Table 2). By contrast, tracks forming trackway F4 are more closely grouped, and the values are somewhat more conservative and consistent along the trackway. In trackway F17 (17 measurements), the D parameter ranges from 48 mm to 13 mm (mean: 31.5 mm, SD±0.88, Table 2). It is noteworthy that the highest values are in the tracks that show evidence of hallux and metatarsal impression marks. Depth in trackway F7 (17 measurements) displays a range of values from 1.7 mm to 22.5 mm (mean = 11.6, SD±0.74, table 2). In trackway F5 (5 measurements), depth ranges from 22 mm to 8 mm (mean = 16.6 mm, SD±0.59, table 2). Trackway F4 (10 measurements) shows a range from 5 mm to 12 mm in depth (mean = 7.8 mm, SD±0.2, table 2).

To quantify this substantial intra-trackway depth and length variations, four graphs for trackways F17, F7, F5 and F4 were built using TL, PL (left Y axis) and depth measurements (right y axis) (Fig. 9).

Figure 9 shows that: a) the pace length (PL) displays some variations, especially in trackways F17 and F7, b) the track length (TL) changes to a lesser extent than the depth parameter, and c) that the depth (D) is the most variable measurement. In particular, in F17, there is considerable variation in depth among the first few tracks, yet track length and pace length remain relatively consistent. Between tracks 17.4 (crossing area 1) and 17.9, TL and D both display a decrease of a 19% and 16%, respectively, while PL increases by 26%. From track 17.9 to 17.14, the PL decreases by 13% and D increases by 42%. From track 17.14 to track 17.17 (crossing area 2), D strongly decreases a 58%, while PL and TL do not show remarkable variations.

Between tracks 7.1 and 7.3, trackway F7 displays an increasing PL with constant D and TL. From track 7.4 to 7.8, PL slightly decreases, while D increases until track 7.7 to then decrease in track 7.8. From this point of the graphic, a remarkable increase in D (92%) is recorded from track 7.8 to track 7.13, but PL increases only by 17%. Finally, from track 7.14 to track 7.17 (crossing area 2), although PL and TL remain averagely constant, D strongly decreases by 43%. Trackway F5 displays a decreasing D (63%) from track 5.1 to 5.5, with an average constant PL and TL. Trackway F4 shows an increasing D from track 4.1 to track 4.7 (58%), a variable TL (23% of variation) and a weakly decreasing PL (6%). From track 4.7 to track 4.10, D decreases quite abruptly (33%), while PL and TL do not display significant variations.

## Discussion

The El Frontal site is an exceptional example of high within-trackway morphological variation. The final morphology of a track is determined by the shape of the track maker’s foot, the dynamics of that foot, and the substrate conditions [4], [47], [48]. Within-trackway morphological variation cannot come from variations in foot anatomy, and therefore must originate from horizontal sediment heterogeneity, differences in limb dynamics, or a combination of the two.

The morphological variation of all tracks (Fig. 6 and Appendix S2) is highly influenced by the depth to which the animal sank. By observing the position of each track in the El Frontal tracksite (Fig. 4) and comparing the graphics with each other, it is noticed that similar depth trends are recorded for F4, F7 and F17, which are located parallel in the tracksite. Among trackway segments 4.6–4.10, 17.11–17.14 and 7.11–7.14, a progressive depth increase is recorded, while among trackway segments 4.5–4.1, 17.15–17.17 and 7.15–7.17 depth decreases. Trackways F17 and F7 differ in PL, TL and D values quite strongly (see Table 2), although they behave similarly along three different intra-trackway segments: between 17.2–17.8 and 7.4–7.8, depth decreases in both trackways, between 17.8–17.14 and 7.8–7.13 depth increases and finally, between 17.14–17.17 and 7.14–7.17 depth strongly decreases in both trackways (72% and 43% respectively). This last zone corresponds to the crossing area 2 (Fig. 4), in which trackways are closely located and, although displaying different absolute values of the parameters (Fig. 9), they present a similar trend in responding to the substrate (depth decrease).

Trackway F5, which crosses the site perpendicular to the other trackways, does not display any intra-trackway variation, or similar trends to those of F4, F7 and F17. Nevertheless, tracks 5.4 and 5.5 decrease depth values when approaching to the crossing area 2, where the general tendency is for tracks to be deeper (eg. F17 and F7).

**Figure 10:**
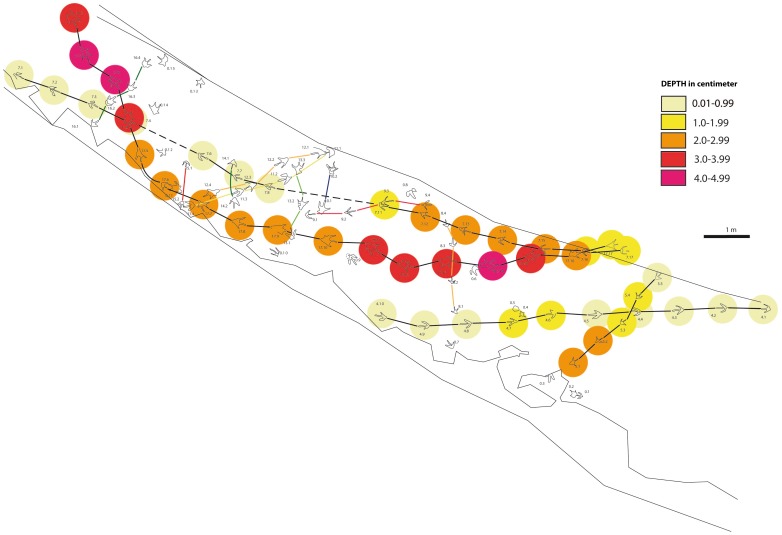
Morphological continuum of the tracks of the El Frontal tracksite. Color scale bar is based on depth intervals.

It has been accepted for a long time that the depth to which a foot sinks is a determinant parameter in understanding the soil mechanics that control track formation [1], [2], [6], [10], [19]–[21], [49]. The deep tracks at the El Frontal site represent part of a continuum that must have been produced on a laterally heterogeneous substrate (Figures 6 and 10). Hence, tracks change their morphology in accordance to their relative position along a substrate consistency gradient that persisted across the site (Figures 9 and 10). Scrivner and Bottjner [50] and Allen [51] suggested that there is a positive correlation between the foot penetration and the degree of deformation in a sediment. At the El Frontal tracksite, the D versus DR and D versus TL graphics (Figures 7 and 8) show a high difference of values for the 49 tracks considered in the sample as a whole and within single trackways. The dispersion graphics underpin the importance of substrate response with respect to track length and depth variations during the indention of the foot. If the substrate conditions of the El Frontal tracksite were uniform throughout the trampled surface, foot loads made by comparably sized animals moving in a dynamically similar fashion (see PL in Fig. 9) would have produced similar tracks (same track length and depth) along single and associated trackways. On the contrary, we observe that track depth and morphology are extremely variable both within a single trackway and the whole track sample (Fig. 9).

Tracks can be used to provide additional information on the conditions of the substrate at the time of track formation. Various works [1], [8], [10], [20], [52]–[54] underscored the fact that substrate properties such as consistency, sediment composition (e.g. proportion of clay minerals), grain size, texture, water content and rate of consolidation control and bias the resulting track morphology. The sedimentological analyses performed on the El Frontal site support with the idea that the original substrate was non-homogenous due to lateral changes in adjoining microfacies. Thin sections of layers 4 and 5 (Fig. 3D–F) reveal sedimentary structures (mud drapes and symmetrical ripples) that are usually found when the surrounding environment is characterized by interruptions in the continuity of water flows, such as the current produced in environments with tidal influence [37], [38]. This implies that the energetic episodes are frequent, fluctuant and intermittent (Fig. 3D–F). A substrate with a higher water content offers more favourable conditions to produce deep tracks (Fig. 3A–C, 6A,B1). A drier substrate of firmer quartz dominant sandstone is more likely to have produced shallow tracks (Fig. 3A–B,6A1,B). The El Frontal tracks exhibit different depths and morphologies resulting from varying rheological conditions due to a lateral facies of changeable consistency, perpendicular to F17, F7 and F4, but parallel to F5, which is affected to a lesser extent.

Finally, in the current state of knowledge it seems difficult to assign any of the studied tracks to a particular group of tridactyl trackmakers, especially regarding the difficulties distinguishing between theropods and ornithopods in the Iberian Range during Berriasian times [33], [55]. The presence of hallux marks and large steps might indicate a probable theropod origin [56], though they are not exclusive characters of this group. Several theropod ichnotaxa have been described in the Huérteles Formation: *Megalosauripus isp.* [57], *Kalohipus bretunensis* [58], “*Fillichnites gracilis*” [59] and *Archaeornitipus meijidei* [60]. Moreover, some grallatorid [61] and *Buckerburgichnus-*like tracks have been reported [62]. Inferences on possible ichnotaxa in the El Frontal tracksite are tangled by the morphological variability observed in the site. The substrate bias in the morphology prevents us from assigning any of the tracks to a particular ichnotaxon, and the strong substrate bias affects track morphology in such a way that it rarely correlates with real foot anatomy of the trackmaker. Interestingly, this study opens a new window into the interpretation of the aforementioned ichnotaxa in the Huérteles Formation and questions whether some of them might represent taphotaxa *sensu* [63].

## Conclusions

The El Frontal tracksite displays a variety of tridactyl track morphologies and provides a valuable example of how track geometry might be dominantly affected by substrate conditions during formation, implying that rheology is the major factor in track formation. The photogrammetry models and depth analyses spotlighted that the deep and shallow tracks are part of a continuum of track morphologies and depths. Sedimentological analyses revealed that the site was a non-homogenous substrate that experienced lateral changes due to fluctuating and intermittent flow episodes in a fluvial-deltaic environment. The tracksite differentiation of substrate consistencies and the vast range of intra-trackway morphologies suggest that tracks were produced by similar trackmakers crossing the lateral gradient of heterogeneous substrate consistencies.

The presented analyses underline the influence of substrate on the final track morphology and length. The within-trackway variation highlights that ichnotaxonomic assignations of sediment-biased tracks should be avoided.

## Supporting Information

Appendix S1 Caption of three-dimensional El Frontal tracksite. Scale bar 1 meter.
(TIF)

Appendix S2 Photogrammetry and depth analysis respectively undertaken with free software VisualSFM and Schlumberger package Petrel of the El Frontal tracksite. Tracks are disposed verticallly to underpin the intra-trackway morphological variation. Color scale green and yellow indicates the track layer, purple is the deepest point recorded for depth and red is the highest point recorded for displacement rims.
(TIF)
