# Supplementary figures and images for: Intra-Trackway Morphological Variations Due to Substrate Consistency: The El Frontal Dinosaur Tracksite (Lower Cretaceous, Spain)
Source: PLoS One. 2014 Apr 3;9(4):e93708. doi: 10.1371/journal.pone.0093708 (PMC3974801; doi:10.1371/journal.pone.0093708)

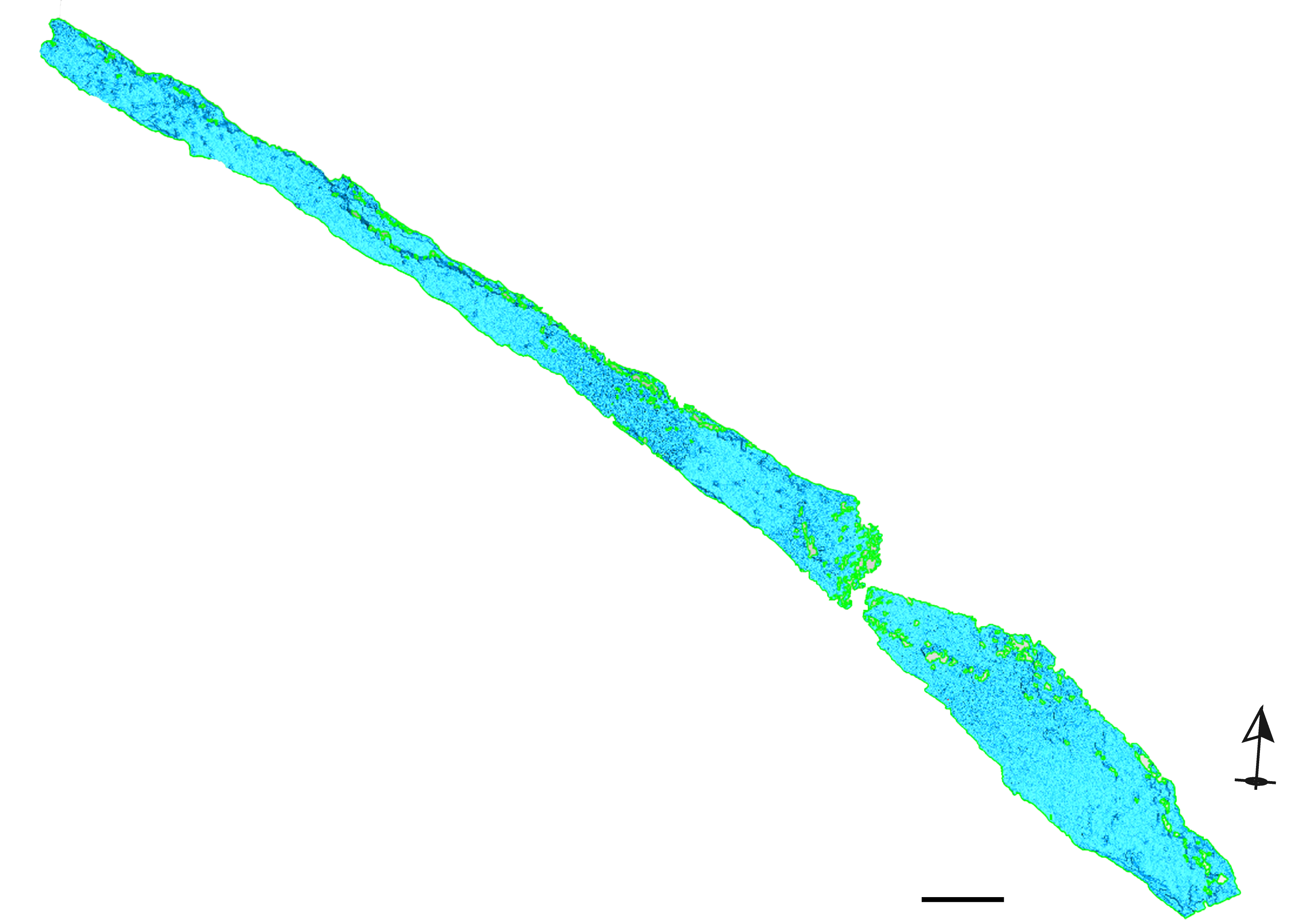

Supplement: Appendix S1 — Caption of three-dimensional El Frontal tracksite. Scale bar 1 meter. (TIF) [file pone.0093708.s001.tif]

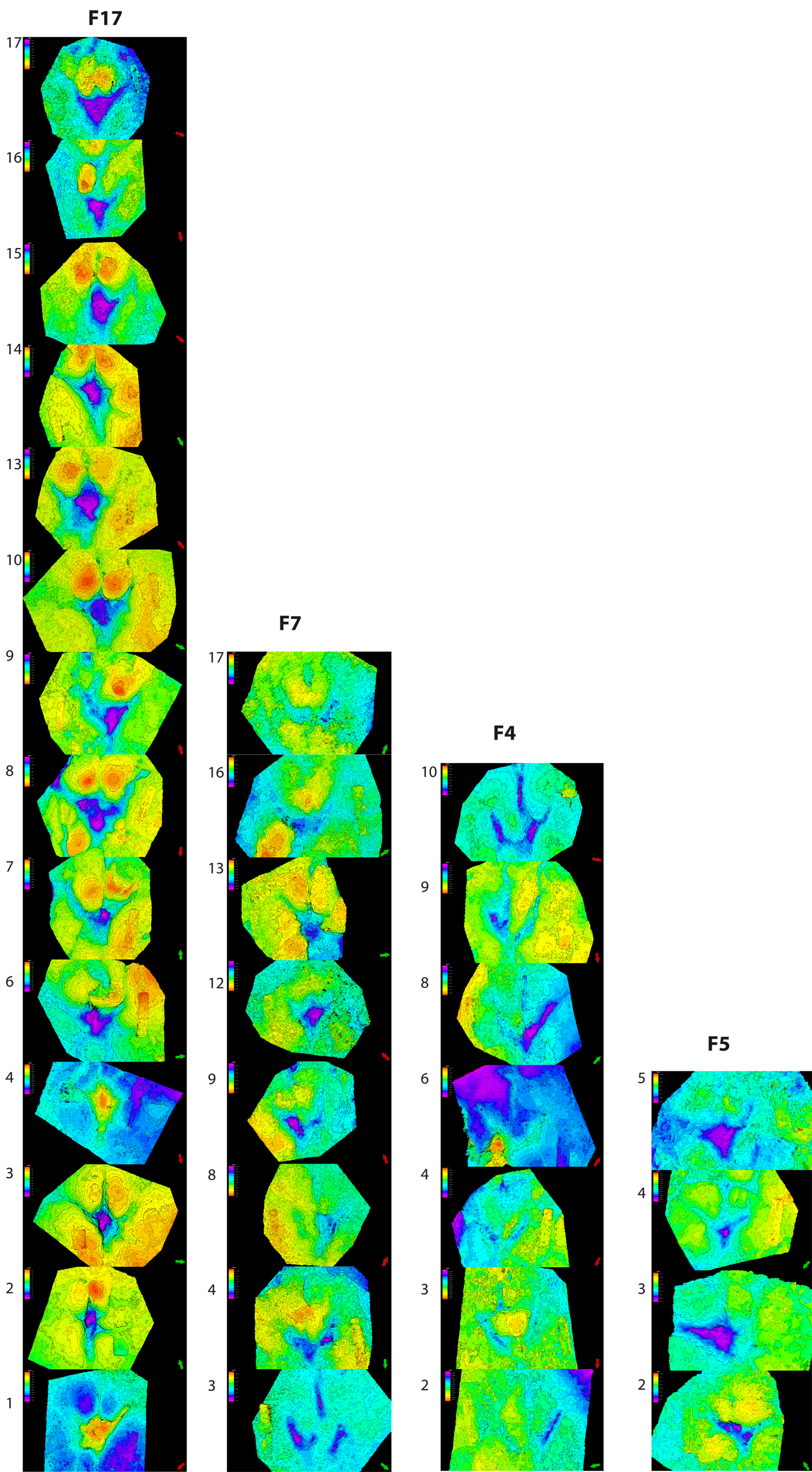

Supplement: Appendix S2 — Photogrammetry and depth analysis respectively undertaken with free software VisualSFM and Schlumberger package Petrel of the El Frontal tracksite. Tracks are disposed verticallly to underpin the intra-trackway morphological variation. Color scale green and yellow indicates the track layer, purple is the deepest point recorded for depth and red is the highest point recorded for displacement rims. (TIF) [file pone.0093708.s002.tif]
